# Risk perceptions and attitudinal responses to COVID-19 pandemic: an online survey in Ethiopia

**DOI:** 10.1186/s12889-021-10939-x

**Published:** 2021-05-25

**Authors:** Zewdie Birhanu, Argaw Ambelu, Diriba Fufa, Mohammed Mecha, Ahmed Zeynudin, Jemal Abafita, Ashenafi Belay, Feleke Doyore, Lemessa Oljira, Endale Bacha, Jilcha Feyisa, Zinabu Hadis, Ketema Ayele, Yohannes Addisu, Birhanu Gutu, Demu Tesfaye, Temesgen Tilahun, Gudeta Imana, Tadele Tolosa, Seblework Mekonen, Yimenu Yitayih, Nega Jibat, Mathewos Moges, Ayinengida Adamu, Abraham Teym, Adamu Kenea, Taffere Addis, Akalework Mengesha, Yohannes Kebede

**Affiliations:** 1 Departemnt of Health, Behavior, and Society, Faculty of Public Health, Jimma University, Jimma, Ethiopia; 2 Department of Environmental Health Sciences and Technology, Faculty of Public Health, Jimma University, Jimma, Ethiopia; 3 Department of Pediatrics and Child Health, Faculty of Medical Sciences, Jimma University, Jimma, Ethiopia; 4 Department of Internal Medicine, Faculty of Medical Sciences, Jimma University, Jimma, Ethiopia; 5 Department of Medical Laboratory Sciences, Faculty of Health Sciences, Jimma University, Jimma, Ethiopia; 6 Department of Economics, College of Business and Economics, Jimma University, Jimma, Ethiopia; 7 Department of English Language and Literature, College of Social Sciences and Humanities, Jimma University, Jimma, Ethiopia; 8 Department of Public Health, Wachemo University, Hossana, Ethiopia; 9 School of Public Health, College of Health and Medical Sciences, Haramaya University, Harar, Ethiopia; 10 Oromia Regional Health Bureau, Risk Communication and Community Engagement Unit, Finfinne, Ethiopia; 11 Department of Oncology, College of Health Sciences, Addis Ababa University, Addis Ababa, Ethiopia; 12 School of Public Health, College of Health Science, Mekelle University, Mekelle, Ethiopia; 13 Department of Public Health, Dire Dawa University, Dire Dawa, Ethiopia; 14 Department of Health, Behavior, and Society, Faculty of Health Sciences and Medicine, Dilla University, Dilla, Ethiopia; 15 Department of Public Health, College of Medicine and Health Sciences, Dambi Dollo University, Dembi Dollo, Ethiopia; 16 Department of Internal Medicine, Adama Hospital Medical College, Adama, Ethiopia; 17 Department of Obstetrics & Gynecology, Institute of Health Sciences, Wollega University, Nekemte, Ethiopia; 18 Department of Internal Medicine, College of Medicine and Health Sciences, Ambo University, Ambo, Ethiopia; 19 School of Veterinary Medicine, College of Agriculture and Veterinary Medicine, Jimma University, Jimma, Ethiopia; 20 Department of Psychiatry, Faculty of Medical Sciences, Jimma University, Jimma, Ethiopia; 21 Department of Sociology, College of Social Sciences, Jimma University, Jimma, Ethiopia; 22 Department of Environmental Health, Hawassa University, Hawassa, Ethiopia; 23 Department of Health System Management and Health Economics, School of Public Health, College of Medicine and Health Sciences, Bahir Dar University, Bahir Dar, Ethiopia; 24 Department of Environmental Health, College of Health Science, Debre Markos University, Debre Markos, Ethiopia; 25 Department of Public Health, Mettu University, Mettu, Ethiopia; 26 Ethiopian Institute of Water Resources, Addis Ababa University, Addis Ababa, Ethiopia; 27 Department of Sociology, College of Social Sciences and Humanities, Wolaita Sodo University, Sodo, Ethiopia

**Keywords:** COVID-19, Coronavirus, Extended parallel process model, Health threat, Risk perception, Perceived severity, Vulnerability, Efficacy, Self-efficacy, Collective efficacy, Attitudinal response, Attitude, Risk communication, Ethiopia

## Abstract

**Background:**

Effective risk communication is one of the critical strategies in the response to COVID-19. This study examined risk perceptions and attitudinal responses to COVID-19 among the educated section of the society in Ethiopia.

**Methods:**

An internet-based survey was conducted from April 22 to May 04, 2020, in Ethiopia. A questionnaire addressing the perception of health threat-combination of perceived vulnerability (PV) and perceived seriousness (PS), and perceived efficacy-combinations of perceived response efficacy (PRE), perceived self-efficacy (PSE), and perceived collective efficacy (PCE). The data were analyzed using SPSS 21.0. Descriptive statistics were computed after the standardization of the scores. The scores for overall efficacy and threat were split by median value and response classifications were generated through threat by efficacy interactions. For statistical significance, 95% CI and *p*-value < 0.05 were used.

**Results:**

A total of 929 respondents submitted their responses. Eight hundred and twenty-eight (89.1%) of the respondents were male and 753 (81.1%) were Christian. The perceived threat to COVID-19 was generally low (median = 58.3). The median score for overall efficacy, PRE, and PSE were 79.8, 87.5, and 80.0, respectively. However, the median value (66.7) for PCE was relatively low. Perceived threat significantly varied by age, education, occupation, and place of residence (*p* < 0.05). Perceived efficacy significantly differed by gender, residence, and use of some sources of information (*p* < 0.05). In terms of response to COVID-19, 290 (31.2%), 239 (25.7%), 175 (18.8%) and 225 (24.2%) of the respondents were in the responsive, pro-active, avoidant, and indifferent attitudinal categories, respectively. The avoidant and indifferent groups constituted a fear control response (mal-adaptive motivation towards COVID-19 protective behavior) whereas responsive and pro-active categories formed a danger control response (self-protective motivation). These responses varied significantly by residence, region, religion, and sources of information (*p* < 0.05).

**Conclusions:**

Understanding people’s perceived health threat and efficacy is a critical step toward creating risk communication campaigns. Hence, this study provided an insight that has the potential to inform the COVID-19 risk communication campaigns targeting the educated section of the society, by ensuring a balanced combination of threat appeals and efficacy messages for improved self-protective responses.

**Supplementary Information:**

The online version contains supplementary material available at 10.1186/s12889-021-10939-x.

## Background

The coronavirus disease outbreak was first found in Wuhan, China in December 2019, when clusters of pneumonia cases of unknown causes were reported to be associated with exposure to seafood [1–3]. On 30 January 2020, the World Health Organization (WHO) declared that the outbreak was a Public Health Emergency of International Concern and on 11 February 2020, WHO declared it a pandemic disease [4]. Globally, as of the middle of May 2020, WHO reported that there were over four million total confirmed cases, and over 300, 000 confirmed deaths [5]. In Africa, a total of 39,087 confirmed cases and 1642 confirmed deaths were reported as of April 30, 2020 [6]. In Ethiopia, the first COVID-19 confirmed case was published on March 13, 2020, and the first COVID-19 confirmed death was recorded on 05 April 2020 [7]. As of May 01, 2020, there were 194 confirmed cases and 4 confirmed deaths due to COVD-19 in Ethiopia, with a transmission scenario classified as “Clusters of cases” [5].

Early evidence documented that the transmissions of COVID-19 were linked to direct exposure to the Seafood in the Wuhan City of China, where animal-to-human transmission was presumed as the main route [3, 8, 9]. However, subsequent evidence has concluded that the virus is transmitted from human-to-human, and symptomatic individuals are the major source of infection to spread. The transmission mostly occurs through respiratory droplets from coughing and sneezing, with the possibility of aerosol transmission in case of protracted exposure to elevated aerosol concentrations in closed spaces [8, 10, 11]. Moreover, evidence indicated that the transmissions are mostly limited to family members, healthcare professionals, and other close contacts within 6 ft or 1.8 m. Owing to the possibility of surface contamination, the transmission may also occur through fomites (inanimate surfaces or objects) in the immediate environment around the infected person [3, 8, 12, 13]. Regarding the duration of contaminated surfaces, the coronavirus can survive on plastic for up to 2–3 days, stainless steel for up to 2–3 days, and cardboard for up to 1 day [11]. Even though an effective cure has not been discovered yet, prompt care-seeking practices enhance recovery from the illness and contribute to combating the spread of the virus. Currently, there are a large number of vaccine candidates under development against coronavirus disease, with promising results [14, 15].

Recent updates indicated that the main signs and symptoms of COVID-19 present at illness onset may include one or more of fever or chills, cough, shortness of breath or difficulty breathing, fatigue, muscle or body aches, headache, loss of taste or smell, congestion or runny nose, nausea or vomiting and diarrhoea [1, 3, 8, 16]. Older men with medical comorbidities are more likely to get infections, with higher mortality rates [17, 18].

COVID-19 affected countries around the world are promoting a comprehensive package of public measures such as hand hygiene, respiratory etiquette, social distancing, use of masks, isolation, and treatment of ill individuals, quarantine of asymptomatic contacts based on the country context, avoiding mass gatherings, school closures and other public health measures such as transportation closures, and/or workplace closures [19]. The WHO suggested that the travel measures and temporary restrictions can be gradually lifted based on thorough risk assessments of the country context and the local epidemiology, the national health and social measures, and the capacities of health systems [20].

In response to the pandemic, Ethiopia has swiftly implemented several public health measures, including partial lockdown to stop the transmission and prevent the spread of the virus (eg. school/university closure, enforcement of social distancing, virtual working policy in some sectors, avoidance of crowded places, restrictions of movements, banned social gatherings promotion of frequent hand washing and respiratory hygiene, closing borders, mandatory 14 days quarantine for international travelers, and also declared a state of emergency [21, 22]. Ethiopian COVID-19 responses also included risk communication and community engagement (RCCE). RCCE is one of the most critical response strategies to educating and actively engaging the community and the wider public in response to COVID-19 to stop the transmission and spread of the virus [23, 24]. Since the first COVID-19 confirmed case recorded in Ethiopia on March 13, 2020, the country has deeply engaged in COVID-19 risk communications activities to inform and educate the public to encourage adherence to protective measures. The public is constantly exposed to different versions of COVID-19 risk communications and promotional messages through different communication channels and sources such as social media platforms, electronic and print media, internet communication, and different community-based educational activities. Even though repetitive risk communication campaigns have been underway, no study has been conducted to examine how the public was perceiving risks and responding to health threats due to COVID-19. Indeed, perceptions and attitudinal responses to the pandemic may change over time due to several factors, such as the magnitude of the problem (eg. disease prevalence, mortality and morbidity levels), and content and coverage of risk communication activities.

### The theoretical basis of the study

The study used the Extended Parallel Processing Model (EPPM) as a guiding framework. EPPM is a communication model focusing on fear arousal and efficacy messages to activate and direct desirable attitudinal responses to initiate behavioral change [25–27]. The EPPM builds on the concept of perceived health threat (a combination of subjective perception of severity and susceptibility) and overall efficacy (a combination of perceived response efficacy and self-efficacy) that lead to message acceptance and, ultimately, desired behavior changes in the population [28, 29]. Thus, EPPM suggests that risk communication campaign messages must contain the appropriate mix of threat arousing messages specifically addressing perceived vulnerability) (PV) (how likely is it that one might contract COVD-19) and perceived seriousness (PS) (how serious are the consequences if one became infected with COVID-19). Additionally, the campaign message should contain efficacy-related components that address perceived response efficacy (PRE) (i.e. Beliefs regarding the effectiveness of the proposed solution such as basic protective measures are effective in reducing personal risk to COVID-19) and perceived self-efficacy (PSE) (i.e., personal belief and confidence in one’s own ability to successfully practice recommended measures, in this case, ability to adhere to COVID-19 basic protective and safety measures) [25–27, 29]. Thus, upon exposure to COVID-19 messages, the individual could be either in the fear control process (developing defense mechanism to campaign messages) or in the danger control process (developing protective motivation response-adopt COVID-19 protective measures).

The degree to which an individual feels threatened by a COVID-19 determines his or her motivation to act, while his/her confidence to effectively avert the threat determines the nature of the action [25, 29]. In clear terms, fear of a health risk (COVID-19 in this case) can cause either adaptive/self-protective behaviors or maladaptive/self-defeating behaviors depending on the level of threat and efficacy. This means that when perceptions of both threat and efficacy are high, individuals practice self-protective behavior [25, 29]. Conversely, when perceptions of a threat are strong, but perceived levels of efficacy are low, the individual develops maladaptive or denial attitudinal responses. Based on the effect of interactions between threat and efficacy, there are four distinct attitudinal groups: (1) responsive (high threat-high efficacy); (2) pro-active (low threat-high efficacy); (3) avoidant (high threat-low efficacy); and (4) indifferent groups (low threat-low efficacy) [26, 30–32]. Each group will respond differently to a given campaign message and thus, need to be addressed with the right combination of threat and efficacy belief messages [25–27, 29, 33, 34]. Consequently, individuals in the responsive category would have an attitude that favors an active adoption of COVID-19 protective measures with strong motivations while those in the pro-active category are believed to practice minimal self-protective response but has a low motivation to try much. On the other hand, avoidant groups are characterized by defense motivation such as denial and counter COVID-19 protective measures and the indifferent group is-even do not process the relevance of the issues. The responsive and pro-active group constitutes a danger control response to COVID-19 which leads to protective attitudes, intentions, and behaviors. On the other hand, fear control responses (i.e. avoidant and indifferent groups) result in various coping mechanisms characterized by defensive avoidance (i.e. denial, being against, risk minimization, risk acceptance, and message rejections) [27, 29, 31, 32, 35]. Even though the EPPM assume that communication factors play a significant role in risk perception and response [26, 27, 29, 35], how people respond to risks may be influenced by many factors including wider socio-cultural norms, contextual and political situations, and individual daily experiences [36–39], educational backgrounds [37–41]. Likewise, peoples’ efficacy to perform the behavior can also be influenced by internal factors such as emotional arousal and external cues through evaluations of resources and conditions needed to carry out the behaviors and perception of collective efforts or interdependence [42–44].

### Aim of the study

Assessing the public response to COVID-19 yields a valid prediction of the community’s preventive practices against the pandemic which will have substantial input to enhance ongoing risk communication and community engagement campaigns. Hence, this study examined risk perceptions and attitudinal responses (focusing on perceptions of threat and efficacy) to COVID-19 among the Ethiopian population that had access to internet services to respond to the online questionnaire survey.

## Methods and materials

### Settings

An internet-based cross-sectional study was conducted in all regions of Ethiopia involving populations who had access to internet connections to respond to the online survey questionnaire on COVID-19 perceptions and behaviors. The online survey was preferred for practical reasons concerning the COVID-19 public health emergency crisis making field data collection impossible. Indeed, an online survey has significant advantages over other formats during the emergency crisis to generate rapid first-hand evidence (speed and timeliness) that supports ongoing public health interventions-provides very good reach and coverage using several online formats such as e-mail and social media sharing. The online survey is most convenient for the respondents to answer the survey questions at a suitable time for themselves and they may take as much time as they need to answer individual questions [45, 46]. Moreover, low administration costs and ease of follow-up are additional values of online surveys [45]. However, an online survey could have some limitations such as perception as junk mail and lack of representativeness of the general population [45, 46].

### Survey designs

The survey participants were invited to take part in the study through different online platforms. The survey tool was created through Google Form and the survey link was promoted through e-mail communications, social media (Facebook and LinkedIn), and the Jimma University website. The questionnaire was designed in a user-friendly layout, with clear answering instructions requiring only a minimum of computer/smartphone skills to navigate around and for their completion. The questionnaire was pre-tested to ensure the adequacy, instructions, and ordering of the questions, comprehensiveness of the contents, and feasibility of the technology. The survey link was shared on April 22, 2020, and the responses were collected until May 04, 2020.

### Measurements

The questionnaire consisted of participants’ demographic profile, source of information, and exposure to COVID-19 messages, health threat (perceived susceptibility and perceived severity), and perceived efficacy (perceived response efficacy and self-efficacy). To measure perceptions of health threat and efficacy, the Risk Behavior Diagnosis (RBD) Scale approach [30, 32] was adapted to the context of COVID-19 taking into account WHO’s recommendations on COVID-19 basic protective and safety measures [19]. The RBD is a Likert –scale type tool that allows rapid assessment of people’s belief and attitudinal response to health risk indicating whether the public is in danger control or fear control processes [26, 30–32]. Specifically, the scale is composed of four measures: threat measures-PV to threat (COVID-19) (4-items) and PS of threat (4-items); and efficacy measures-PSE (12-items) and PRE (10-items). PSE items were addressing personal confidence to practice COVID-19 self-protective measures and PRE was measuring personal beliefs in the effectiveness of the recommend COVID-19 protective/safety/precautions measures in reducing threat or infections. All the items were stated on a five-point Likert scale ranging from strongly disagree [1] to strongly agree [5].

### Operationalization measure of RBD scale

Principal component analysis (PCA) with Varimax rotation method was conducted to explore and validate the RBD subscale dimensions. Informed by previous methodologies [47, 48], indices were produced by summing up its respective items and rescaled to (0–100) value for standardization and comparison of the scales using $\mathrm{Y}=\frac{\left(X- Xmin\right)\mathrm{n}}{Xrange\ }$ where Y is the adjusted variable, X is the original variable, Xmin is the minimum observed value on the original variable and Xrange is the difference between the maximum score and the minimum score on the original variable and n is the upper limit of the rescaled variable. First, we computed a separate composite score for each construct (PV and PS) and the median value was calculated from the composite score separately after the score adjusted to 100%. To produce overall threat and efficacy score, we first summed up perceived vulnerability and perceived seriousness to produce threat score and similarly efficacy subscales summed up to yield an overall efficacy score. Then, the threat score and overall efficacy score were rescaled (adjusted) to 0–100 value for comparisons, which were then used to compute an overall median value for perceived threat and perceived efficacy separately. Based on the median split [26, 29, 35, 49], the efficacy and threat scores were classified as low and high and group attitudinal response classification (response quadrant) was made by threat-overall efficacy interactions as responsive (high threat, high efficacy), avoidant (high threat, low efficacy), pro-active (low threat, high efficacy) and indifferent-no-responses (low threat, low efficacy). Responsive and pro-active were in danger control process whereas avoidant was in fear control process while indifferent category characterized by lack of response at all-did not consider COVID-19 as being real or relevant to them and often not even aware of threat (COVID-19) [26, 27].

### Data analysis

The online response submitted by respondents was transferred into an excel database and exported to SPSS version 21.0 for analysis. Respondents’ background variables are presented in frequency tables; mean and median score was computed for each sub-scale of the threat and efficacy measures. T-test and one-way-ANOVA are computed to compare mean differences by selected background variables. To examine the relationship between perceived health threat and perceived efficacy measures in explaining how they interact to produce the desired response, the Pearson correlation coefficient was used and the chi-square test was used to assess the association between attitudinal response categories and selected background characteristics. A 95% confidence interval and a *p*-value less than 0.05 are used to determine a statistically significant association. To account for diversity in respondents’ backgrounds, the analysis was segregated by selected background characteristics.

## Results

### Demographic profile of participants

In this online survey, a total of 929 participants responded to the questionnaire. Table 1 presents the background information of the survey respondents. Accordingly, the majority (50.8%) of the respondents were in the age range of 30–39 years followed by 18–29 years of age groups, accounting for 185(30.7%). In terms of gender, the majority (89.1%) of the survey respondents were male. Even though more than half (56.6%) of the respondents were from the Oromia national regional state, there were responders from all regions of Ethiopia. Concerning the educational level, more than half, 536 (57.7%) of the respondents were holders of master’s degrees.

**Table 1:** Demographic characteristics of respondents, May 2020, Ethiopia

Variables	Response category	Frequency	Percentage
Age in years	18–29	285	30.7
	30–39	472	50.8
	> = 40	172	18.5
Gender	Male	828	89.1
	Female	101	10.9
Marital Status	Single	308	33.2
	Married	592	63.7
	Others^a^	29	3.1
Religion	Orthodox	417	44.9
	Protestant	336	36.2
	Muslim	114	12.3
	Others	62	6.7
Place of residence	Zonal level town	520	56.0
	Big towns (regional/national/capitals)	319	34.3
	District /semi-urban/rural	90	9.7
Educational status	University/college degree	259	27.9
	Second/masters degree	536	57.7
	PhD/equivalent	134	14.4
Main occupational category	Health sectors	209	22.5
	Educational institution	501	53.9
	NGO	58	6.2
	Student	72	7.8
	Others	89	9.6
Region	Tigray	49	5.3
	SNNP	103	11.1
	Oromia	526	56.6
	Amhara	52	5.6
	Addis Ababa	139	15.0
	other regions	60	6.5

^a^widowed, divorced, in a relationship

#### Exposure to COVID-19 messages and source of information

**Fig. 1:**
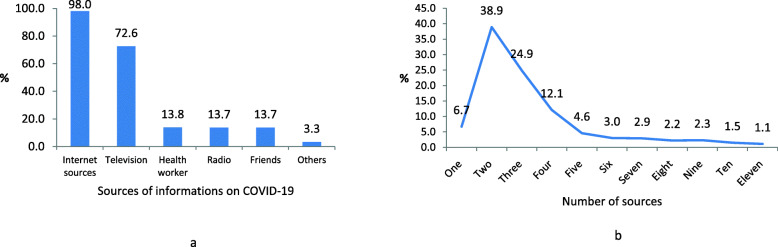
Source of information about COVID-19, May 2020. Internet sources were the major source of information about COVID-19 followed by Televisions stations (**a**)

All of the participants (100%) replied that they have heard of the coronavirus disease (COVID-19). In Fig. 1a, the sources of information about COVID-19 are shown, and Fig. 1b presents the number of sources respondents were exposed to obtain information. Accordingly, for almost all of them (98.0%), the major source of information about COVID-19 was a wide range of internet platforms (such as broadband cable, Wi-Fi, mobile data, mobile wireless, digital subscriber line) followed by Television (72.6%). Only a few respondents (13.8%) were received information from health workers, radio, friends, and other sources (Fig. 1a). On the other hand, respondents were mostly received COVID-19 related information from multiple sources, ranging from one-to-eleven sources. Consequently, 38.9 and 24.9% of the respondents were exposed to two sources while only 6.7% were exposed to a single source of the message (Fig. 1b).

#### Awareness of symptoms of COVID-19

**Fig. 2:**
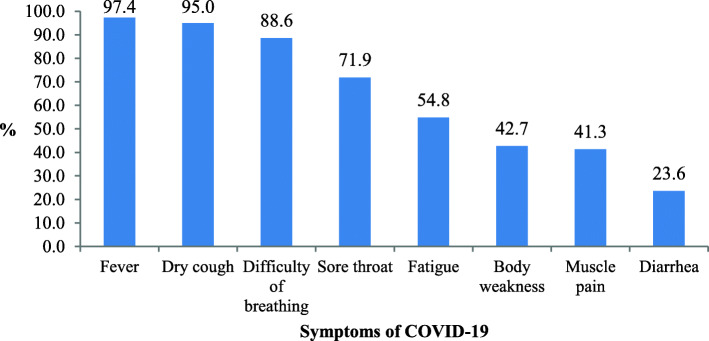
Knowledge of symptoms of COVID-19, Ethiopia, May 2020

The survey revealed that many respondents were accurately identified the common symptoms of COVID-19. Accordingly, the most frequently reported symptoms included fever (97.4%), dry cough (95.0%), and difficult breathing (88.6%) (Fig. 2).

#### A perceived threat to COVID-19: perceived vulnerability (PV) and perceived seriousness (PS)

The RBD scale of threat and efficacy were subjected to PCA and the initial analysis indicated that the measures gave rise to six components which jointly explained 56.8% of the variance. However, to improve the interpretations and retain only meaningful items in the component, two items that were related to PS (1-item) and PV (1-item) were removed from the model. Then, the analysis was repeated where the final PCA explained 55.9% of the variance with five components that aligned to the concept of threat and efficacy in response to COVID-19. The first factor was related to PRE (personal belief of the effectiveness of recommended COVID-protective measures) and it explained 19.6% of the variance and the second factor which is named PSE explained 14.5% of the variance. Another underlying dimension of measure of efficacy was related to the collective efficacy or ability of a member of society to control over the protective measures. This factor was named perceived collective efficacy (PCE) and it explained 7.6% of the variance. Other dimensions, namely PV to COVID-19 and PS of COVID-19 explained 7.9 and 6.3% of the variance, respectively (Table 2).

**Table 2:** Factor loading for PCA of RBDS on COVID-19, May 2020, Ethiopia

Items	Components					% A&SA	
	PRE	PSE	PV	PCE	PS	Yes (%)	95%CI for Yes (%)
Avoiding crowded places and close contact with anyone prevent the risk of infection with COVID-19.	0.826					97.4	96.4–98.4
Avoiding touching eyes, nose, and mouth prevents infection with COVID-19.	0.792					95.9	94.6–97.2
Maintaining social/physical distancing prevents the risk of infection with COVID-19.	0.775					95.8	94.5–97.1
Covering your cough/sneezing using the bend of your elbow or a tissue prevents the spread of COVID-19.	0.773					96.3	95.1–97.5
Staying at home help to prevent infections with COVID-19.	0.711					95.6	94.3–96.9
Isolation and treatment of people who are infected with the COVID-19 are effective ways to reduce the spread of the virus	0.665					97.0	95.9–98.1
Staying informed and following advice given by your healthcare provider can reduce the chance of acquiring COVID-19	0.660					97.1	96.0–98.2
Following good respiratory hygiene is effective to protect the people around you from COVID-19	0.563					89.5	87.5–91.4
Washing hands frequently with soap and water or using alcohol-based hand rub kills the virus that causes COVID-19	0.557					90.1	88.2–92.0
For fever, cough, and difficulty breathing, seeking medical care early help to manage COVID-19	0.534					96.3	95.1–97.5
I have the skill to follow the recommended hand washing practices to prevent myself from COVID-19.		0.701				96.6	95.4–97.7
I can always cover my cough using the bend of my elbow or a tissue to prevent the spread of COVID-19.		0.693				90.0	88.1–91.9
I am confident that I can wash my hands frequently with soap and water or using an alcohol-based hand rub to keep myself from COVID-19		0.687				91.0	89.1–92.8
I can avoid touching my eyes, nose, and mouth to prevent infection with COVID-19		0.609				86.0	83.8–88.2
I have the resource (water, soup) to wash my hands frequently with water and soap to prevent myself from COVID-19.		0.584				87.0	84.8%-89.1
By following good respiratory hygiene I can protect the people around me from COVID-19		0.573				85.5	83.2–87.7
I can be stay informed and follow the advice given by the health care provider.		0.545				94.2	92.7–95.7
The use of personnel protective equipment is effective to prevent COVID-19 infections		0.490				90.2	88.3–92.1
It is likely that I am at risk of getting a COVID-19 infection			0.784			52.4	49.2–55.6
I will likely get a COVID-19 infection			0.706			41.4	38.3–44.6
In many aspects, I am less likely to acquire COVID-19			0.694			51.8	48.6–55.0
It is possible that I will get a COVID-19 infection			0.669			74.3	71.5–77.1
I am confident that I can stay at home easily to prevent COVID-19				0.758		52.4	49.2–55.6
I am confident that I can avoid crowded places and close contact with anyone to protect myself from COVID-19.				0.675		79.0	76.4–81.6
I can maintain at least a 2-m distance between myself and anyone to prevent infection with COVID-19.				0.634		77.7	75.0–80.4
I am confident that Ethiopia can win the battle against the COVID-19 virus				0.516		53.1	49.9–56.3
I believe that COVID-19 is extremely harmful					0.799	79.9	77.3–82.5
I believe that COVID-19 has serious negative consequences on my life					0.718	68.8	65.8–71.8
I believe that COVID-19 infection is a severe disease					0.697	78.4	75.7–81.0
**% of Variance explained (total = 55.9%)**	**19.6**	**14.5**	**7.9**	**7.6**	**6.3**		

*PRE* Perceived Response Efficacy, *PSE* Perceived Self Efficacy-personal level, *PV* Perceived Vulnerability, *PCE* Perceived Collective Efficacy, *PS* Perceived Severity/Seriousness, *A&SA* Agreed and Strongly Agreed

### Item-based analysis

For simplicity and utility, the items in each final sub-scale were collapsed into Yes (agree and strongly agree) and No (disagree, strongly disagree, and neither agree and disagree), and the result is presented in Table 2. Accordingly, the response to each PRE item was quite high, with the lowest 89.5% and as high as 97.4%. Of the PRE items, the most relevant ones were avoiding crowded places and close contacts (factor loading = .826), avoiding touching eyes, nose, and mouth (factor loading = 0.792), and maintaining physical distancing (factor loading =0.775). Likewise, a close examination of individual items for PSE was found to be high among respondents, ranged between 85.5 and 96.6% of the respondents believed that they had the skill to follow recommended hand washing practices to prevent themselves from COVID-19. However, lack of confidence in PCE such as staying at home (52.4%), avoiding crowded places (79.0%), and maintaining at least 2-m physical distancing (77.7%) and confidence in Ethiopia to battle the COVID-19 virus (53.1%) were observed. In contrast, only 52.4% (95%CI: 49.2–55.6%) of the respondents agreed/strongly agreed with the vulnerability statement, “I am at risk for getting COVID-19 infection.” Similarly, only 68.8% (95%CI: 65.8–71.8%) of the respondents agreed/strongly agreed with the statement “I believe that COVID-19 has serious consequences on my life”.

#### Descriptive statistics for efficacy and threat scales and sub-scales

The mean and median scores for perceived threat and perceived efficacy scales and sub-scales are summarized in Table 3. Thus, the mean score for overall perceived health threat was 56.6 ± 15.2-and for PV it was found to be 49.3 ± 15.4 with a median value of 50.0. In contrast, the overall mean (79.3 ± 13.1) and median (79.8) value of the perceived efficacy score was relatively higher-except for PCE (63.9 ± 20.7).

**Table 3:** Descriptive statistics for Efficacy and threat sub-scales, May 2020

Measurement Scales	Mean (SD)	Median (IQR)
**Perceived Threat (overall)**	56.6 (15.2)	58.3 (20.8)
Perceived Seriousness (PS)	72.3 (22.6)	75.0 (33.3)
Perceived Vulnerability (PV)	49.3 (15.4)	50.0 (14.3)
**Efficacy (overall)**	79.3 (13.1)	79.8 (17.9)
Perceived Response Efficacy (PRE)	85.4 (13.8)	87.5 (20.0)
Perceived Self-Efficacy (PSE)	79.5 (14.9)	80.0 (16.7)
Perceived Collective Efficacy (PCE)	63.9 (20.7)	66.7 (26.7)

#### Correlation analysis of efficacy and threat perceptions

**Fig. 3:**
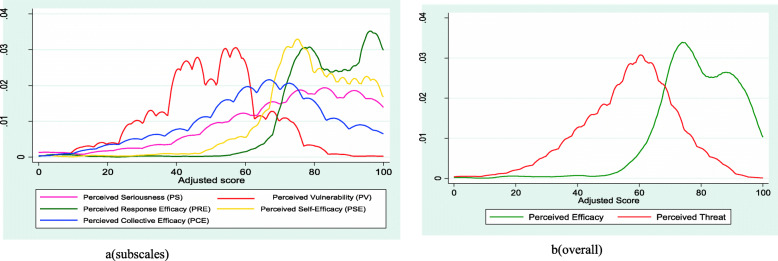
Kernel density estimation to visualize the shape and nature of relationship among the measures of perceived threat and perceived efficacy, May 2020, Ethiopia. With exception of perceived collective efficacy (PCE), all measures of efficacy were consistently higher whereas measures of health threat-specially perception of vulnerability was very low (**b**). Overall health threat and efficacy were also show clear difference between perception of threat (very low) and efficacy (.e. perceptions of the effectiveness of the COVID-19 preventive measures and beliefs one’s own ability to exert personal control to perform protective behavior) (**b**)

Except for the correlation between PCE and PV, which is very weak negative (*r* = − 0.077, *p* < 0.05), all the sub-scales were significantly positively correlated to each other (*p* < 0.05). Overall, the perceived threat was also positively correlated to perceived efficacy (an increase in perceived threat also correlated with an increase in overall efficacy), but a very weak correlation (*r* = 0.203, *p* = 0.001) (Table 4). The shape and nature of the relationship among the measures of the perceived threat and perceived efficacy are visualized in Fig. 3 using the kernel density estimation. The estimation shows significant gaps between the constructs believed to interact together to produce the desired response to COVID-19.

**Table 4:** Pearson correlation coefficient for scales

Scales	Efficacy overall)	Threat (overall)	PS	PV	PRE	PSE	PCE
Efficacy (overall)							
Perceived Threat (overall)	0.203^a^						
PS	0.228^a^	0.811^a^					
PV	0.058	0.676^a^	0.119^a^				
PRE	0.884^a^	0.256^a^	0.252^a^	0.116^a^			
PSE	0.905^a^	0.169^a^	0.185^a^	0.053	0.702^a^		
PCE	0.666^a^	0.022	0.092^a^	−0.077^b^	0.345^a^	0.519^a^	

^a^ Correlation is significant at the 0.01 level (2-tailed)

^b^ Correlation is significant at the 0.05 level (2-tailed)

### Variations of perceived threat

The analysis of mean difference revealed that mean perceived threat (*p* = 0.02) and PS (*p* = 0.038) was significantly varied by age (decreased mean score as age increased), but the PV did not significantly different by age (*p* > 0.05). Moreover, mean PV was significantly different by the use of mobile data, with a higher mean value among respondents who had access to the internet service (mean = 50.6 vs 47.7, *p* = 0.004). Similarly, the mean value for an overall health threat was significantly different by the use of mobile data (mean = 57.9 vs 54.9, *p* = 0.002) though the PS did not vary by use of mobile data (*p* > 0.05). Indeed, the mean PS was significantly higher among respondents who were using the Wi-Fi internet source (mean = 73.4 vs 68.5, *p* = 0.006). Correspondingly, the mean value for PS (*P* = 0.020), PV (*P* = 0.023), and overall health threat (*p* = 0.004) was significantly lower among respondents with higher education levels. Nevertheless, the mean value for overall health threat and its sub-scales (PV and PS) did not significantly vary by gender, religion, marital, and sources of information such as social media, TV, radio, health workers, friends, broadband internet, Wi-Fi, home-based network, and health workers (*p* > 0.05). Region-wise (Table 5), only PV (*p* = 0.020) (lowest in Tigray, moderate in Oromia, and highest in other regions) and PRE (*p* = 0.042) significantly varied. Concerning occupational categories, the mean value for overall health threat (*p* = 0.005) and PV (*p* = 0.001) was significantly higher among workers in health sectors whereas the mean value for PS (*p* = 0.020) and overall threat (*p* = 0.012) were significantly highest in district/semi-urban areas.

**Table 5:** Perception mean score of a threat to COVID-19 and efficacy of protective measure by region, May 2020

Regions	Perceived Threat	Perceived Efficacy (overall)	PRE	PSE	PCE	PS	PV
Amhara	55.0	80.3	88.0	79.2	63.2	67.3	51.0
Oromia	55.9	78.2	84.1	78.7	62.8	71.5	48.8
Addis Ababa	56.3	81.3	87.1	82.1	65.7	71.3	49.6
Tigrai	56.5	80.4	86.4	80.5	65.7	78.2	44.0
SNNP	58.3	79.7	86.0	78.2	67.2	74.8	50.1
Other regions	61.5	81.6	88.3	82.2	64.0	76.5	54.2
Total	56.6	79.3	85.4	79.5	64.0	72.3	49.3
F-test	1.870	1.954	2.314	1.742	1.105	2.054	2.681
P-value	0.097	0.083	0.042	0.122	0.356	0.069	0.020

#### Variations of perceived efficacy

**Fig. 4:**
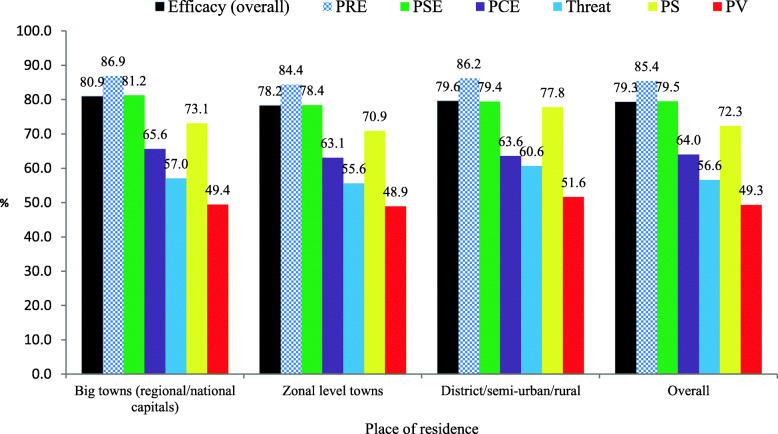
Perceptions of a threat to COVID-19 and efficacy of protective measure by residence, May 2020

**Fig. 5:**
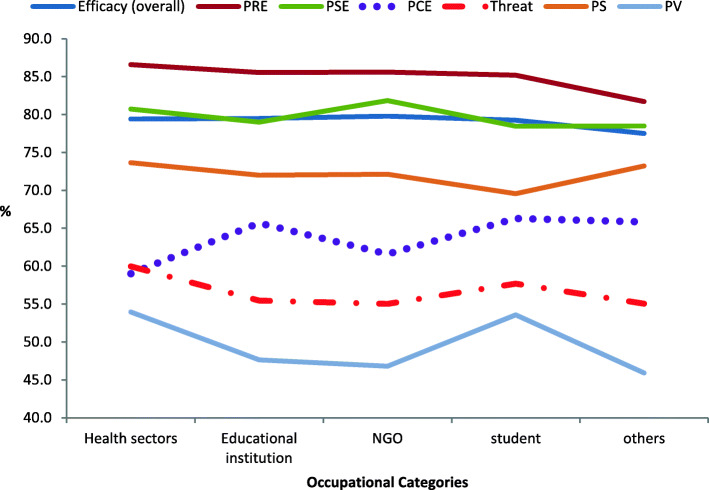
Perceptions of a threat to COVID-19 and perceived efficacy of protective measure by occupational categories, May 2020

The mean score for perceived efficacy (overall) and all of its sub-scales (PRE, PSE, and PCE) did not vary by age, marital status, education, and region (*P* > 0.05). The mean score for an overall efficacy (*p* = 0.010), PRE (*p* = 0.015), and PSE (*p* = 0.041) were significantly higher among females but the mean score for PCE did not vary by gender (*p* > 0.05). The mean score of threat and efficacy measures by place of residence is shown in Fig. 4, where the mean score for perceived response efficacy (PRE) was consistently high across the place of residence; lies between 84.4 and 86.9, whereas the mean score for the PV was consistently low (ranged from 48.4 to 51.6) across the place of residence. Likewise, the mean score for overall efficacy (*p* = 0.016) and PSE (*P* = 0.029) were highest in a big town but lowest at the zonal level whereas PRE (*p* = 0.031) was also highest in big towns but lowest at district levels. Figure 5 displays the mean score of a perceived health threat to COVID-19 and the perceived efficacy of protective measures by occupational categories. Perceived response efficacy was consistently high across occupational categories and all the measures, except PCE (*p* = 0.001) which was significantly lowest among respondents working in the health care setting, were not significantly different across occupational categories.

Except for PSE, overall efficacy (*p* = 0.004), PRE (*p* = 0.005), and PSE (*p* = 0.008) were higher among people using the official website for a source of information. Similarly, the mean overall perceived efficacy (*p* = 0.003), PRE (=0.009), PSE (*p* = 0.010), and PCE (*p* = 0.031) was significantly higher among respondents who used health workers as a source of information about COVID-19. In addition, PCE was higher among user of radio (*p* = 0.011), own internet at home (*p* = 0.006), TV users (*p* = 0.046), broadband internet users (*p* = 0.046) and Wi-Fi users (*p* = 0.017). However, mean perceived efficacy did not vary by the exposure to multiple sources of information such as mobile data, social media, friends (*p* > 0.05).

#### Classifications of attitudinal response-effects of threat by efficacy interactions

To explore the state of danger control and fear control process, interaction scores representing four response categories (quadrants) were generated by interacting threat and efficacy measures, yielding responsive, pro-active, avoidant, or indifferent responses. The result is presented in Table 6. Accordingly, 290 (31.2%) of the respondents were in a responsive reaction to COVID-19 and hence, in danger control process-taking protective action against COVID-19. The third class (quadrant III) was pro-active respondents (lesser amount of danger control-taking some protective actions, but lack the motivation to try much) accounting for 175 (18.8%) of the quadrants. The second class (Quadrant II) which constituted 239 (25.7%) was avoidant respondents. These are groups of respondents in fear control reactions typically in a state of denial about COVID-19 and responding against it and indifferent respondents (no response) accounted for 225 (24.2%) of the study participants.

**Table 6:** Effects of threat by efficacy interaction to produce danger control and fear control responses

Perceived threat	Perceived efficacy		
	High Efficacy	Low Efficacy	Total
High Threat n(%)	Quadrant I: Responsive(Danger Control) 290 (31.2%)	Quadrant II: Avoidant (fear control) 239 (25.7%)	529 (56.9%)
Low Threat n (%)	Quadrant III: Pro-active (small danger control) 175 (18.8%)	Quadrant IV: Indifferent (No response) 225 (24.2%)	400 (43.0%)
**Total n (%)**	**465 (50.1%)**	**464 (49.9%)**	**929 (100)**

The response categories were significantly varied by region (x^2^ = 37.301, *p* = 0.001), religion (x^2^ = 24.223, *p* = 0.004), place of residence (x^2^ = 19.334, *p* = 0.004), use of Wi-Fi (x^2^ = 9.422, *p* = 0.024), health workers (x^2^ = 10.538, *p* = 0.015) and official website (x^2^ = 12.260, *p* = 0.007) for source information regarding COVID-19 (Table 7).

**Table 7:** Associations of attitudinal response to COVID-19 with demographic characteristics, Ethiopia, May 2020 (*N* = 929)

Characteristics	Response classifications membership					X^2^, *P*-value
	Indifferent n (%)	Avoidant n (%)	Pro-active n (%)	Responsive n (%)	Total n(%)	
**Place of residence**						
Zonal level town	146 (28.1)	134 (25.8)	94 (18.1)	146 (28.1)	520 (56.0)	19.334, 0.004
District /Semi-urban/rural	14 (15.6)	33 (36.7)	13 (14.4)	30 (33.3)	90 (9.7)	
Regional capitals	65 (20.4)	72 (22.6)	68 (21.3)	114 (35.7)	319 (34.3)	
**Region**						
Oromia	140 (26.6)	151 (28.7)	92 (17.5)	143 (27.2)	526 (56.6)	37.301, 0.001
Addis Ababa	28 (20.1)	32 (23.0)	34 (24.5)	45 (32.4)	139 (15.0)	
SNNP	26 (25.2)	21 (20.4)	14 (13.6)	42 (40.8)	103 (11.1)	
Amhara	10 (19.2)	10 (19.2)	17 (32.7)	15 (28.8)	52 (5.6)	
Tigray	10 (20.4)	12 (24.5)	13 (26.5)	14 (28.6)	49 (5.3)	
Other regions	11 (18.3)	13 (21.7)	5 (8.3)	31 (51.7)	60 (6.5)	
**Religion**						
Orthodox	94 (22.5)	94 (22.5)	89 (21.3)	140 (33.6)	417 (44.9)	24.223, 0.004
Protestant	95 (28.3)	77 (22.9)	58 (17.3)	106 (31.5)	336 (36.2)	
Muslim	22 (19.3)	43 (37.7)	18 (15.8)	31 (27.2)	114 (12.3)	
Others^a^	14 (22.6)	25 (40.3)	10 (16.1)	13 (21.0)	62 (6.7)	
Source of information						
**Health Worker**						
Yes	24 (18.8)	24 (18.8)	26 (20.3)	54 (42.2)	128 (13.8)	10.538, 0.015
No	201 (25.1)	215 (26.8)	149 (18.6)	236 (29.5)	801 (86.2)	
**Internet official website**						
Yes	36 (23.4)	24 (15.6)	33 (21.4)	61 (39.6)	154 (16.6)	12.260, 0.007
No	189 (24.4)	215 (27.7)	142 (18.3)	229 (29.5)	775 (83.4)	
**Wireless-Wi-Fi**						
Yes	51 (24.6)	37 (17.9)	46 (22.2)	73 (35.3)	207 (22.3)	9.422, 0.024
No	174 (24.1)	202 (28.0)	129 (17.9)	217 (30.1)	722 (77.7)	

Note: The attitudinal response classes did not significantly vary by age, gender, education, marital, source of information (social media, TV, radio, friends), and occupation categories (*p* > 0.05). ^a^Wakeffeta, Adventists

## Discussion

This study examined attitudinal responses to the COVID-19 pandemic in educated sections of the Ethiopian population through an online questionnaire survey based on EPPM as a guiding framework. Accordingly, it revealed that the study populations were in a state of a low perceived health threat to COVID-19, but developed optimal PRE (i.e. believe that an effective response is available to reduce risk of COVID-19) and PSE (believed that they were capable to utilize the response to reduce the risks). However, low perceived threat (mainly, perceptions of low possibility to acquire COVID-19) combined with inadequate PCE affected respondents’ self-protective motivations to minimize the risk of COVID-19. More specifically, perceived threat to COVID-19was generally low in the study populations reflecting that large portions of the public did not have the belief that COVID-19 is relevant and consequential to them. Most importantly, the PV (the belief that I am at risk for a COVID-19) was quite low, indicating that people were not accurately perceived progressive sense of susceptibility to the disease. A telephone-based study conducted in Ethiopia also reported that the level of risk perception was quite low where only 31.1% of the respondents perceived that they were at risk of coronavirus [43]. Another study also reported very low levels of-risk perceptions [50]. Indeed, risk perception is a complex process greatly influenced by many factors including, but not limited to individuals’ beliefs and perceptions, wider socio-cultural system, environmental and political conditions, geographic locations, contextual factors, and individual daily experiences [36–39].On the other hand, habitual engagement in high-risk activities (eg. attending crowded places, not practicing respiratory and hand hygiene), but yet free of COVID-19 can lead to higher risk tolerance and lower risk perception [38] which might be the case in the present context.

To successfully provoke a positive attitudinal response to COVID-19, the public must accurately perceive that the COVID-19 is a serious health condition impacting their life in multiple ways and also must have a strong belief that one is personally susceptible to it at any time and in any locality. There was strong evidence that with a low level of perceived threat appeal, the public might not develop the right cognitions such as positive intentions, and attitude that mediates positive behavioral change [31, 51, 52]. The low level of COVID-19 threat among the respondents might be suggesting that the ongoing public campaigns had a deficit in threat appeal content especially in addressing dimensions of vulnerability to and severity of the COVID-19. This may require that the risk communication campaign may have to evaluate campaign message contents to carefully augment perceptions of susceptibility and vulnerability claims using personalized messages narratives, storytelling, and the use of real-life stories from COVID-19 patients who share similar characteristics with the target audience [53–55]. Additionally, local communication resources, community groups, and networks can be utilized for localized educational activities. In all cases, it is essential to provide credible evidence that threats are real and likely even in communities where there are no confirmed COVID-19 cases, yet. On the other hand, COVID response teams should be careful when communicating to the public as some of the messages may be counterproductive. For instance, reporting COVID-19 incidence in a given (eg. zero incidence or case) community; zero incidence during house to house terminal screening of households; telling people COVID-19 is flue like illness, and assuring that most people recovering from it without needing medical treatment could harm people’s perceptions of threat-interpreted as an insignificant threat.

The analysis indicated that the overall health threat to COVID-19 and its sub-scales (perceptions of vulnerability and severity) did not significantly vary by gender, religion, marital status, and sources of information. However, a higher perception of vulnerability and threat was significantly associated with the use of mobile data and Wi-Fi internet as a source of information about COVID-19. This suggests the need to work with telecommunication services to increase access to cellular networks, especially in remote areas. Perception of severity and overall threat significantly associated with age decreased as age increase, but PV did not vary by age which is contrary to the expectations-evidence indicates that elderly people are at higher risk of COVID-19 [2, 8, 12]. Of course, the proportion of elderly people presented in the current study was few which might be affected the stability of statistical analysis. On the other hand, increased educational levels were negatively associated with a decreased threat which could be due to people with higher education levels might have adequate resources needed to practice protective measures to avert the threat. Even though the evidence is insufficient in the context of COVID-19, there are abundant data which confirmed that education plays a key role in influencing how people perceive and respond to health risk [37–41]. This is because risk perception is related to cognitive skill, ability to use health-related information, and health knowledge, better informed or educated individuals, are more likely to develop risk perceptions risk factors, making educational gradients robust perceived risk predictors [38, 39, 41, 56]. Perception of vulnerability also varied by regions where it was lowest in the Tigray region and moderate in Oromia which might have to do with differences in risk communications practice, the difference in settings, and confirmed case distribution. On the other hand, the overall health threat was significantly highest among respondents who work in the health care sector, and respondents living in district/semi-urban settings. Indeed, evidence suggests that risk perceptions and perceptions of threat may be influenced by contexts, settings, individuals’ daily experience, and other several factors which might be valid in this context as well [36–38]. Certainly, health workers are the frontline fighters of COVID-19 and it is not a surprise if they experienced a high level of health threat. However, health workers need special attention as a high threat could lead to frustration, psychosocial problems, and poor adherence to protective measures [29, 51].

In this study, respondents demonstrated a high level of overall efficacy across settings and demographic factors-large number of the respondents held strong beliefs that COVID-19 protective behaviors were effective enough or efficacious to avert risks and they also largely believed that they certainly practice the recommended measures. In specific terms, response efficacy (subjective perceptions of the effectiveness of recommended measures) was sufficiently high compared to an earlier study, in Ethiopia, which reported 65 and 68%, PCE, and PSE, respectively [57]. This may suggest that the risk communication campaigns were somewhat successful in achieving public trust about the effectiveness of COVID-19 preventive measures. Nevertheless, it should be noted that peoples’ efficacy can also be influenced by internal cues such as the degree of emotional arousal and external cues such as the amount of resources required (eg. access to protective equipment) to carry out the behaviors [42]. A lack of protective materials was also reported in another survey in Ethiopia [43]. Evidence has widely documented that high efficacy conditions energized adaptive coping behavior [27, 31, 35, 49, 51]. On the other hand, PSE (peoples’ confidence) also matters in the realization of behaviors-people will drop into a defensive or denial attitude, if the perceived ability to carry out the recommendations is low despite high PRE.

Interestingly, PSE to exercise COVID-19 preventive measures had two dimensions–self-efficacy related to protective behaviors relatively under personnel locus of control (hand hygiene, respiratory hygiene, avoiding touching eyes, and noses, and use of personal protective equipment) and collective efficacy regarding behavioral practices that are relatively outside the control of an individual, needing external influences and cooperation such as maintaining physical distancing, avoiding crowded places, and staying at home. This study indicated that people had weak confidence to practice protective measures related to social activities, suggesting the need to include high PCE message-especially targeting physical distancing, avoiding crowded places, and how to stay home. This can be done by elaborating, demonstrating, and addressing local-specific barriers to these protective measures. Recent reports also indicated that behavioral practice related to PCE was quite low indicating how hard the behavior was for the people to adhere to [43, 57]. Existing evidence also indicated that the extent to which people believe that other people are also cooperative or act in an interdependent way towards the recommended actions influences people’s efficacy, especially collective efficacy [44].

This study also revealed that measures of overall efficacy (PRE, PSE, and PCE) did not vary by age, marital status, and education, regional areas but females had significantly higher efficacy. This suggests that females were more responsive to COVID-19 with better self-confidence to practice COVID-19 protective behaviors. On the other hand, belief in the effectiveness (PRE) of COVID-19 protective behaviors was significantly varied by place of residence and occupational categories and as such tailored and local specific communication interventions are needed to address the specific needs and gaps. Interestingly, the use of the official website and health workers as a source of COVID-19 related information contributed to the development of self-efficacy including for PCE. Thus, it is important to strengthen the use of official websites and health workers to boost people’s confidence in adherence to COVID-19 protective measures. Even though the mechanism was not clear, the use of radio, home-based internet service, broadband internet services, Wi-Fi users tended to have better confidence to adhere to physical distancing, avoiding crowded spaces.

Based on the premise of threat by efficacy interaction, four distinct attitudinal response categories, namely danger control categories (responsive and pro-active) and fear control categories (pro-active and indifferent respondents), splitting the respondents nearly to 50–50% (self-protective behaviors vs mal-adaptive or self-defeating behaviors). This has significant practical implications to COVID-19 risk communication program since nearly half of the study population was in fear management (defense motivations to campaign messages), characterized by undesirable attitudinal responses such as denial and rejections of prescribed public health measure, and failure to considering COVID-19 be real or relevant to their life. Thus, risk communication efforts are required to seriously revise messaging approaches and content of messages, by emphasizing threat appeal messages while advancing the people’s confidence in the effectiveness of the recommended measures and their belief in their ability to exercise them. Pieces of evidence suggest people develop mal-adaptive or engaged in self-defatting behaviors when both threat and efficacy are low, or threat is high at a low efficacy level [25, 27, 29, 58]. To motivate people towards self-protective responses, risk communication programs should be informed by studies in designing message contents, formats, and appeals that are appropriate to build balanced perceptions of health threat and efficacy belief in the target audience [59]. Communication researches suggest that involving influential and credible sources either as a messenger or source of messages can increase the effectiveness of persuasive health messages [55, 59] that the COVID-19 risk communication designers may adopt. It is also crucial to adapt the communication messages to the local context and specific audience segments, especially by residence, settings, main occupational categories, demographic factors such as gender. Simultaneously, it is important to note that each attitudinal response segment will respond differently to particular messages and thus, need to be addressed with different health messages and strategies [25–27, 29, 33, 34]. The EPPM assumes that individuals take time to appraise threat and efficacy. These appraisals are assumed to happen continuously, and once the levels of perceived threat or efficacy reach certain thresholds (critical points), subsequent responses are triggered [27, 29, 31, 49, 51]. Hence, it is vital to regularly monitor how the public is responding to the COVID-19 campaign.

### Strengths and limitations of the study

As with all internet-based surveys, this study has several advantages [45, 46] and perhaps, the only practical method to rapidly generate evidence that assists the ongoing public health emergency responses to COVID-19. The study is the first of its kind in Ethiopia in examining attitudinal response to COVID-19 and could have implications for the COVID-19 risk communication program targeting literate adult population groups in Ethiopia. Even though the COVID-19 risk perceptions may cluster by literacy status of the populations, the present findings could have implications for the general adult populations as educated individuals are part of the broader society and would share common risk factors when it comes to COVID-19 transmission and spread. Yet, an aggregated risk communication approach based on the literacy level of the populations would be helpful. Nevertheless, this survey exhibits limitations of any internet-based survey-respondents were only educated and those who had access to internet connections, representation by demographic factors (such as sex, age, and religious groups) and geographic distributions couldn’t be achieved thereby affecting the representativeness of the general populations. To this end, females were less represented in this study which might be reflected that females had less access to internet services in Ethiopia or less willing to respond to an online survey.

## Conclusions

People’s perceived risk- perceptions about their susceptibility to COVID-19 and how severe it is combined with perceptions of efficacy would play an important role in motivating people towards self-protective behaviors. For protective behaviors to occur, people should experience a sufficient subjective threat to COVID-19 with a high level of personal and collective efficacy to adopt and maintain appropriate self-protective practices. Deficiencies in one of the perceptions could lead to a mal-adaptive or self-defeating attitudinal response. The present study documented an early indication that the level of people’s perception of threat to COVID-19 was generally low in the study population with a substantial gap in perceptions of vulnerability. However, the level of perceived effectiveness of COVID-19 protective measures was so strong among the participants though people still lack collective efficacy in some of the COVID-19 protective measures leaving half of the study population in fear control responses which characterized by counter-productive behavior that involves the use of cognitive defense mechanisms to manage the state of their fear instead of adopting appropriate COVID-19 protective measures. This could create a conducive behavioral and social atmosphere for COVID-19 to spread easily. Thus, COVID-19 risk communications activities targeting an educated crowd in Ethiopia are needed to focus on communicating health risk messages that combine balanced fear appeals (moderate threat provoking messages) and efficacy messages to build people’s collective and personal efficacy to carryout COVID-19 recommended prevention measures. Given that the level of perceived vulnerability was quite low, a risk communication program must be laid an adequate emphasis on communicating health messages that build people’s perceptions of vulnerability to COVID-19. It is essential to include health risk messages that can provoke higher perceived personal vulnerability to COVID-19, and messages that can build a sense of concerns and worries. A personalized messaging approach like ‘you likely get COVID-19 if you are not exercised a comprehensive self-protective measure’, could help to enhance subjective perceptions of vulnerability. In doing so, it is vital to adapt the basic COVID-19 protective measures and messages to the local context, and audience needs and profile through the use of credible and trusted sources such as health workers and experts in the field. While the use of mass media platforms is essential to create a broader understanding and also useful to provoke threat perceptions, it is important to complement it with localized community education and engagement approaches such as the use of community volunteers, frontline health workers, and different community groups and networks (eg. women-centered development army and self-help groups). Risk communication programmers should also utilize social media platforms (especially Facebook) in regulated ways to disseminate only verified messages, updates with an effective and prompt feedback system. Regular or periodic surveys of public perceptions of threat and efficacy will be necessary to monitor and adjust the content and format of COVID-19 health risk communications efforts.

## Supplementary Information

**Additional file 1.**

## Data Availability

The datasets during and/or analyzed during the current study are available from the corresponding author on reasonable request.

## Abbreviations

COVID-19: Coronavirus Disease 19
EPPM: Extended Parallel Process Model
IQR: Interquartile Range
PCA: Principal component analysis
PCE: Perceived Collective Efficacy
PE: Perceived Efficacy
PSE: Perceived Self-efficacy PRE Perceived Response Efficacy
PV: Perceived Vulnerability
RBD: Risk Behavior Diagnosis
WHO: World Health Organization
