# Supplementary material for: Risk perceptions and attitudinal responses to COVID-19 pandemic: an online survey in Ethiopia
Source: BMC Public Health. 2021 May 25;21:981. doi: 10.1186/s12889-021-10939-x (PMC8148408; doi:10.1186/s12889-021-10939-x)
Supplement: Supplementary file 1 — Additional file 1. [file 12889_2021_10939_MOESM1_ESM.pdf]

Jimma University, Institute of Health

Questionnaire to assess knowledge, community perceptions, responses and practice towards COVID-19

Dear Sir/Madam,
Jimma University, Institute of Health conducting a nationwide rapid assessment to assess knowledge, attitude, practices and responses to COVID-19 diseases among scholars who are supposed to take leading role in combating the spread- academic staffs, health professionals and other civil servants. The study would generate useful information and insight on scholars and community's perceived awareness and extent of self-care practices and responses to communication messages. The finding will help to reshape and make further adaptations of ongoing efforts to prevent the spread of COVID-19 virus. Therefore, we kindly request your honest and kind response to this survey questionnaire. Indeed, your participation is voluntarily. But we highly value participation and contribution at this critical time. It may take 20-30 minutes to fill the question. Your responses will be completely anonymous. If you have any questions regarding this research, contact Zewdie Birhanu (PhD, phone:+251917025852, email: zbkoricha@yahoo.com, Prof Argaw Ambelu, phone: *+251(0)911 82 62 18, email:* aambelu@yahoo.com_)

We would like to thank you for kindly consenting to participate in this survey.
Please click the link to access the questionnaire

Part I: Background characteristics

1. What is your age in completed years? ________________
2. What is your sex? A. Male ☐ B. Female ☐
3. What are your current educational qualifications?
   1) ☐ Primary (grade 1-8)
   2) ☐ High school (grade 9-12)
   3) ☐ Diploma or Level I-IV
   4) ☐ First Degree (BSc/BA)
   5) ☐ Medical Doctor-GP
   6) ☐ Residents (specify year of residency) _____________
   7) ☐ Master degree
   8) ☐ Ph.D
   9) ☐ Clinical Specialty /sub-specialty
   10) ☐ Other (specify)___________________________________
4. What is your current marital status?
   1) ☐ Single (unmarried)
   2) ☐ Married
   3) ☐ Divorced
   4) ☐ Widowed
   5) ☐ Engaged (in relationship)
5. What is your main occupation?
   1) ☐ Merchant/shop

2) ☐ Teacher-elementary /high school
3) ☐ University-Academic
4) ☐ University-administrative staff
5) ☐ Health worker in government facilities (specify professional category)________________
6) ☐ Health worker in private facilities (specify professional category)___________________
7) ☐ Health worker in administration/office (specify professional category)________________
8) ☐ Private work/business
9) ☐ Police/security
10) ☐ Government employ (Ethiotelcom, airlines, bank/insurance/transportations)
11) ☐ work in NGOs
12) ☐ Military
13) ☐ House wife
14) ☐ Daily labouer
15) ☐ Farmer
16) ☐ Student
17) ☐ No job
18) ☐ Private firm/company
19) ☐ Other (specify)__________________________

6. Where is your place of residence?
1) ☐ Urban
2) ☐ Rural

7. In which region of Ethiopia, are you currently living?
1) ☐ Addis Ababa
2) ☐ Oromia
3) ☐ Tigray
4) ☐ Ahmara
5) ☐ Somali
6) ☐ Affar
7) ☐ Benishangul Gumuz
8) ☐ Gambella
9) ☐ Harari
10) ☐ Dire Dawa
11) ☐ Southern nation nationalities and peoples

8. What is your religion affiliation?
1) ☐ Orthodox
2) ☐ Muslim
3) ☐ Protestant
4) ☐ Wakeffeta
5) ☐ Other (please specify)____________________

9. Which internet connection do you mostly use to get information about COVID-19?
1) ☐ Broadband-cable

2) ☐ Cellular-data(telle)
3) ☐ DSL (home private)
4) ☐ Wireless-Wifi
5) ☐ Do not use internet

Part II: Exposure to information

10. Have you heard COVID-19 disease? 1. Yes ☐ 2. No ☐

11. What is your main source of information about COVID-19 disease?
    1) ☐ Internet-official websites
    2) ☐ Television
    3) ☐ Radio
    4) ☐ Friends
    5) ☐ Internet-social media (Facebook, twitter, YouTube etc)
    6) ☐ Health workers
    7) ☐ Neighbors

12. Which source of information about COVID-19 do you most trust? (select two sources you most trust)
    1) ☐ Government Medias (TV/Radio/newsletter etc)
    2) ☐ Private Medias (TV/Radio/newsletter etc)
    3) ☐ Religious leaders
    4) ☐ Local source (announcement, posters, banners/brochures)
    5) ☐ National source (ministry of health, Ethiopian public health institute)
    6) ☐ International media
    7) ☐ Social Medias (Facebook/whatsApp/Telegram/ etc)
    8) ☐ Health Activists (Public figures/etc)
    9) ☐ Health facilities/professionals
    10) ☐ Other (please specify________________________

13. What are the main clinical symptoms of COVID-19? (Multiple response is possible)
    1) ☐ Fever
    2) ☐ Fatigue
    3) ☐ Dry cough
    4) ☐ Muscle pain
    5) ☐ Sore throat
    6) ☐ Diarrhea
    7) ☐ Difficulty breathing
    8) ☐ Body weakness
    9) ☐ Other (specify)___________________________________

Part III: Perceptions and response to COVID-19 messages

For following questions, please indicate (tick) your personal level of agreement with each statement about severity of COVID-19	Indicate your level of agreement by making "X" under category				
	Strongly disagree	Disagree	Neither agree nor disagree	Agree	Strongly agree
14. I believe that COVID-19 infection is severe disease	☐	☐	☐	☐	☐
15. I would rather die than getting COVID-19 infection	☐	☐	☐	☐	☐
16. I believe that COVID-19 has serious negative consequences on my life	☐	☐	☐	☐	☐
17. I believe that COVID-19 is extremely harmful	☐	☐	☐	☐	☐
18. It is likely that I will get COVID-19 infection	☐	☐	☐	☐	☐
19. I am at risk for getting COVID-19 infection	☐	☐	☐	☐	☐
20. It is possible that I will get COVID-19 infection	☐	☐	☐	☐	☐
21. In many aspects, I am less likely to acquire COVID-19	☐	☐	☐	☐	☐
For the following questions, please indicate your personal level of agreement regarding the effectiveness of the actions in reducing risk of COVID-19 infections					
22. Washing hands frequently with soap and water or using alcohol-based hand rub kills the virus that causes COVID-19	☐	☐	☐	☐	☐
23. Maintaining social distancing can prevent infection with coronavirus	☐	☐	☐	☐	☐
24. Avoiding touching eyes, nose and mouth prevent infection with coronavirus	☐	☐	☐	☐	☐
25. Covering your cough/sneezing using the bend of your elbow or a tissue prevent spread of coronavirus	☐	☐	☐	☐	☐
26. Avoiding crowed places and close contact with anyone prevent infection with coronavirus	☐	☐	☐	☐	☐
27. Staying at home help to prevent infections from infection with coronavirus	☐	☐	☐	☐	☐
28. Following good respiratory hygiene is effective to protect the people around you from COVID-19	☐	☐	☐	☐	☐
29. Isolation and treatment of people who are infected with the COVID-19 are effective ways to reduce the spread of the virus	☐	☐	☐	☐	☐
30. Staying informed and following advice given by your healthcare provider can reduce the chance of acquiring COVID-19	☐	☐	☐	☐	☐
31. For fever, cough and difficulty breathing, seeking medical care early help to manage COVID-19	☐	☐	☐	☐	☐
For the following questions, please indicate your personal level of agreement regarding your personal confidence or ability to practice/implement the safety and preventive measures recommend to keep yourself from COVID-19					
32. I am confident that I can wash my hands frequently with soap and water or using alcohol-based hand rub kills the virus for COVID-19	☐	☐	☐	☐	☐
33. I have the skill follow recommended hand washing to prevent myself me from COVID-19.	☐	☐	☐	☐	☐
34. I have the resource (water , soup) to wash my hands frequently with water and soup to prevent myself me from COVID-19.	☐	☐	☐	☐	☐
35. I am confident that I can stay at home easily to prevent COVID-19 .	☐	☐	☐	☐	☐
36. By following good respiratory hygiene I can protect the people around me from COVID-19	☐	☐	☐	☐	☐
37. I can be stay informed and follow advice given by your healthcare provider	☐	☐	☐	☐	☐
38. I am condiment that I can avoid crowed places and close contact with anyone prevent infection with coronavirus	☐	☐	☐	☐	☐
39. I am confidence that Ethiopia can win the battle against the COVID-	☐	☐	☐	☐	☐

19 virus?					
40. I am confident COVID-19 will finally be successfully controlled in Ethiopia	☐	☐	☐	☐	☐
41. Use of personnel protective equipment is effective to prevent COVID-19 infections	☐	☐	☐	☐	☐
42. I can always cover my cough using the bend of my elbow or a tissue prevent spread of coronavirus	☐	☐	☐	☐	☐
43. I can avoid touching my eyes, nose and mouth to prevent infection with coronavirus	☐	☐	☐	☐	☐
44. I can maintain at least 1 metre distance between myself and anyone to prevent infection with coronavirus	☐	☐	☐	☐	☐
